# Erratum

**DOI:** 10.1111/jdi.14058

**Published:** 2023-07-14

This erratum corrects the following:

Ryotaro Bouchi, Tatsuya Kondo, Yasuharu Ohta *et al*. A consensus statement from the Japan Diabetes Society: A proposed algorithm for pharmacotherapy in people with type 2 diabetes. *J Diabetes Investig.* 2023; 14: 151–164. https://doi.org/10.1111/jdi.13960

The below statement should have been included in the original version of this article:

This article is the English version of the “A consensus statement from the Japan Diabetes Society: A proposed algorithm for pharmacotherapy in people with type 2 diabetes” (https://doi.org/10.11213/tonyobyo.65.419) released in Japanese on Volume 65 Issue 8, 2022, on the official website of the Japan Diabetes Society, and has been jointly published in Diabetology International (the official English journal of the Japan Diabetes Society: https://doi.org/10.1007/s13340-022-00605-x) and Journal of Diabetes Investigation (the official journal of AASD).

The online article has been corrected.

The publisher apologizes for the error.
